# Dietary patterns and their association with breast milk macronutrient composition among lactating women

**DOI:** 10.1186/s13006-020-00293-w

**Published:** 2020-06-05

**Authors:** Zhi Huang, Yu-ming Hu

**Affiliations:** 1 Hunan University of Medicine, No. 492 Jinxi South Road, Huaihua, 418000 Hunan China; 2 Department of Toxicology, Hunan Provincial Center for Disease Control and Prevention, Changsha, 410005 Hunan China

**Keywords:** Dietary patterns, Breast milk, Lactating women, Macronutrient, Principal component analysis

## Abstract

**Backgroud:**

Breast milk is the optimal food for infant growth and development. The purpose of this study was to evaluate the association between breast milk macronutrient composition with dietary pattern among lactating women.

**Methods:**

A total of 220 lactating women from 2011 to 2012 in Changsha, a city of south-central China, was recruited using a multi-stage sampling method. Breast milk was collected, and the protein, fat, lactose, total dry matter, and energy contents of breast milk were measured. A 24 h recall method on three consecutive days was used to collect the dietary information of lactating women and an exploratory factor analysis was performed was to identify dietary patterns. The association between the concentration of a breast milk component and dietary pattern was assessed using a multivariable linear regression model.

**Results:**

Three major dietary patterns were classified. Lactating women with dietary pattern 1 mainly ate fresh vegetables and fresh legumes. Those with dietary pattern 2 mainly ate red meat, cereals and eggs, and those with dietary pattern 3 mainly ate fungi and algae, dries legumes and soy milk. Pattern 2 was positively associated with the concentration of protein (B = 0.07, 95% CI 0.00, 0.15), total dry matter (B = 0.20, 95% CI 0.02, 0.38) and energy (B = 1.66, 95% CI 0.03, 3.30) in breast milk. Morever, lactation period was negatively associated with the protein and total dry matter concentrations and positively associated with lactose.

**Conclusions:**

The results show the lactation period was an important factor affecting milk composition and a dietary pattern with high intake of red meat, cereals, and eggs was associated with higher protein, total dry matter, and energy contents in breast milk. These findings show that the dietary patterns of lactating women can affect breast milk macronutrient composition and provide a foundation for improving child health.

## Background

Breast milk is the optimal food for infant growth and development. According to the World Health Organization, mothers should breastfeed infants exclusively for at least 6 months [1]. Infants benefit from breastfeeding, both in the short-term in terms of a reduced incidence and mortality rate of infectious diseases and in the long-term in terms of a reduced risk of adult obesity, diabetes, cardiovascular disease, and other metabolic diseases [2–4].

Human milk contains all the energy, macronutrients, and micronutrients required for infant growth and development during the first 4 to 6 months of life. It also provides various immunological factors and bioactive components [2, 5]. Lactation is a critical period in terms of nutritional needs. Nutrients are required to maintain the mother’s health, infant growth and health, and postpartum recovery [6, 7]. During lactation, the macronutrient (i.e. proteins, carbohydrates, and lipids) content of human milk has large variability, which is weakly associated with the maternal diet [8]. The fatty acid composition of breast milk and its association with the maternal diet during lactation are also relevant [9–11].

Most studies have analysed the association of individual nutrients or foods with the levels of breast milk components. However, dietary intake is a combination of multiple nutrients and foods. Considering the potential for interactions between total dietary intake and foods or nutrients, dietary pattern analysis is widely used to evaluate the impact of diet on health [12–14]. Therefore, by collecting 220 breast milk samples and the dietary data of the donors in south-central China, we analysed the association of breast milk macronutrient composition (i.e. protein, fat, and lactose), total dry matter, and energy with dietary patterns among lactating women to provide a basis for further improvement of maternal and child health.

## Methods

### Study design and population

**Fig. 1:**
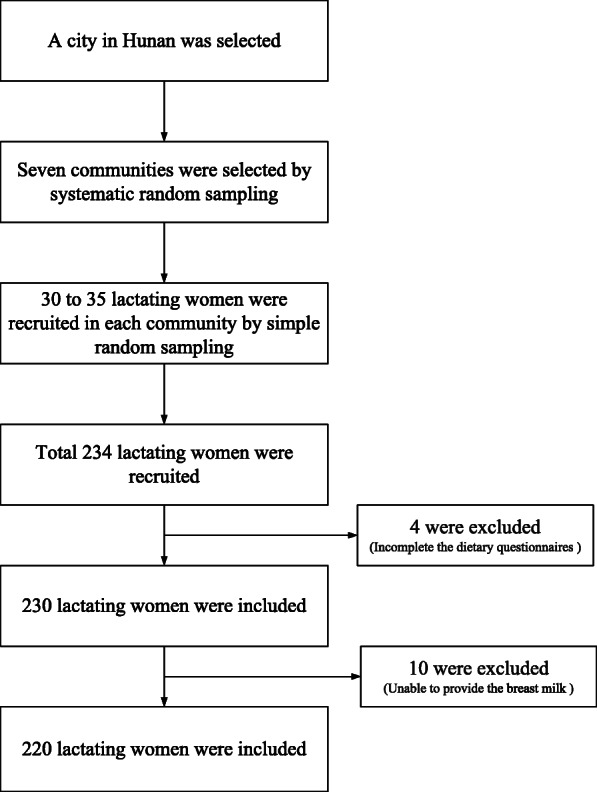
The flow chart of the sampling process

Breast milk samples and dietary data were collected from 2011 to 2012 in Changsha, in Hunan Province, south-central China. This city has an integrated maternal and children’s health system that offers many free primary care services in local community health centres where the lactating women were recruited for our study using a multi-stage sampling method (see Fig. 1). First, considering the gross domestic product (GDP) of the nine districts of Changsha in the past year, Yuelu district with an intermediate GDP was chosen as a representative sample. After ranking all of the communities in Yuelu by income, seven communities were selected by systematic random sampling. Lastly, all of the lactating women in each community were ranked according to lactation period. In our study, the lactation period of women was consistent with the age of their infants; therefore, only the lactation period was analysed. In each community, 30 to 35 lactating women were selected by simple random sampling in the local maternal and children’s health system. The inclusion criteria were lactating women (1) 18 to 40 years old; (2) continuing to breastfeed; (3) with self-evaluated good health; (4) no drinking alcohol or smoking; (5) single pregnancy with a term infant; (6) infant in good health; and (7) informed consent provided. Lactating women with mastitis, infectious diseases, cardiovascular diseases, metabolic diseases, nervous system diseases or cancer, and those who were unable to complete the dietary surveys or provide breast milk samples, were excluded. All lactating women were interviewed by trained interviewers in the Hunan Provincial Centre for Disease Prevention and Control and gave informed consent.

### Milk collection and composition analysis

Breast milk (at least 20 mL) was collected from 8 a.m. to 11 a.m. using an electric breast pump provided by our staff. The protein, fat, lactose, total dry matter, and energy contents of breast milk were measured within 4 h of sampling by Miris HMA, a breast milk analyser (MIRIS, Sweden). The protein, fat, lactose, and total dry matter contents of breast milk were expressed as g/100 mL, and kcal/100 mL for energy.

### Dietary assessment

A 24 h recall method on three consecutive days was used to collect the dietary information of lactating women. Participants were asked to recall their detailed food intake and the estimated portion sizes over the previous three consecutive days using local weight units (1 Liang = 50 g). A total of 246 foods was identified in this study, and these were classified into 23 categories based on the similarity of ingredients and the Chinese Food Composition Table [15] (Additional file 1). The questionnaires used to collect dietary data were administered in interviews by trained investigators (Additional file 2).

An exploratory factor analysis was performed to identify dietary patterns by performing a principal component analysis as described by Xu et al. [16]. The intake of 23 food groups was included in the factor analysis. The dietary patterns were determined by the eigenvalues (> 1), screen plots, factor interpretability, and variance explained. The factor loadings are equivalent to a correlation between a food item and the dietary pattern. The foods with factor loadings of > 0.2 were considered to significantly contribute to the identified dietary pattern. Each participant was assigned a pattern-specific factor score for each dietary pattern, which was calculated as the sum of the products of the factor-loading coefficients and the standardized daily intake of each food associated with that pattern. A higher factor score in each pattern indicated that the participant was more strongly aligned with this dietary pattern. Factor scores were categorized into four quartiles, where Q1 indicates that the dietary pattern was weakly related to the dietary pattern and Q4 indicates that the dietary pattern was strongly related to the dietary pattern.

### Other related variables

Sociodemographic information was collected using questionnaires, and comprised the age of lactating women, lactation period, parity (primipara and multipara), delivery mode (natural, forceps, and caesarean delivery), education level (junior or below, senior, and college or above), occupation (housework and other), and family economic status. Lactation period was subdivided into six groups (7 or fewer, 8–15, 16–30, 31–90, 91–180, and 181+ days) according to the frequency distribution of all samples. Based on the per capita annual income of urban households in Hunan in 2013 [17], the total annual family income was subdivided into low (< 50,000 yuan), intermediate (50,000–100,000 yuan), and high (> 100,000 yuan) groups.

### Statistical analysis

The statistical analysis was performed using Statistical Product and Service Solutions 13.0. The data are expressed as numbers and percentages for categorical variables. The mean (SD) was used to express continuous variables with normally distributed data, and the median (IQR) to express skewed data. The concentration of breast milk components was compared according to the characteristics of the lactating women by ANOVA. Correlation analysis with Spearman’s correlation test was used to determine the correlations between lactation period and breast milk composition. Significant differences between the breast milk macronutrient composition concentrations and dietary patterns across four quartiles were assessed by ANOVA. The association between the concentration of a breast milk component and dietary pattern was assessed using a multivariate linear regression model. Significant predictors (*P* <  0.1) of breast milk macronutrient composition in bivariate analyses were included as factors in the multivariate linear regression model. B with 95% CI was calculated to determine the strength of associations. *P* <  0.05 was considered indicative of statistical significance.

## Results

In total, 234 lactating women were invited to take part in this survey. Participants with incomplete dietary data (*n* = 4) were excluded, as were those who were unable to provide breast milk samples (*n* = 10). After exclusion, 220 lactating women were included in the analysis. The median lactation period of the subjects was 46 (IQR 14–163) days, and their mean age was 27 ± 3 years. Table 1 lists the characteristics and concentrations of breast milk macronutrients in the lactating women. Of the lactating women, 22.27% had lactation periods over 181 days, 44.09% were 26 to 30 years old, and 79.00% were primipara. More than half of the mothers had caesarean deliveries (56.82%), and housework as an occupation (63.64%). Moreover, 40.91% of the mothers had a high-school senior educational level, and 46.82% had a family of middle-level economic status. The mean concentrations in breast milk were 1.37 ± 0.73 g/100 mL for protein, 3.20 ± 1.43 g/100 mL for fat, 6.51 ± 0.40 g/100 mL for lactose, 12.00 ± 1.51 g/100 mL for total dry matter, and 62.43 ± 13.32 kcal/100 mL for energy.

**Table 1:** The characteristics and concentration of breast milk among lactating women in south-central China (number of participants and percentages; *N* = 220)

General characteristic	***N*** (%)
Lactation period	
7 and below days	31 (14.09)
8 to 15 days	34 (15.45)
16 to 30 days	34 (15.45)
31 to 90 days	32 (14.55)
91 to 180 days	40 (18.18)
181 ~ days	49 (22.27)
Age	
25 and below years	87 (39.55)
26 to 30 years	97 (44.09)
31 ~ years	36 (16.36)
Parity	
Primipara	174 (79.00)
Multipara	46 (21.00)
Delivery mode	
Natural	89 (40.45)
Forceps	6 (2.73)
Cesarean	125 (56.82)
Education	
Junior or below	48 (21.82)
Senior	90 (40.91)
College or above	82 (37.27)
Occupation	
Housework	140 (63.64)
Others	80 (36.36)
Family economic level	
Low	81 (36.82)
Middle	103 (46.82)
High	36 (16.36)
Breast milk composition	
Protein (g/100 ml) Mean (SD)	1.37 (0.73)
Fat (g/100 ml) Mean (SD)	3.20 (1.43)
Lactose (g/100 ml) Mean (SD)	6.51 (0.40)
Total dry matter (g/100 ml) Mean (SD)	12.00 (1.51)
Energy (g/100Kal) Mean (SD)	62.43 (13.32)

Table 2 shows the association of the characteristics among lactating women with breast milk composition. There were significant differences in the protein (F_0.05, (5, 214)_ = 25.80, *P* <  0.001), fat (F_0.05, (5, 214)_ = 3.06, *P* = 0.01), lactose (F_0.05, (5, 214)_ = 10.81, *P* <  0.001), total dry matter (F_0.05, (5, 214)_ = 7.42, *P* <  0.001), and energy (F_0.05, (5, 214)_ = 3.49, *P* = 0.01) contents of breast milk during the lactation period. The highest contents were at seven and fewer days for protein, 8–15 days for fat, total dry matter, and energy, and over 181 days for lactose. Correlation analysis showed that the lactation period was negatively correlated with the contents of protein (r = − 0.79, *P* <  0.001), total dry matter (r = − 0.28, *P* < 0.001), and energy (r = − 0.16, *P* = 0.02) in breast milk, but was positively correlated with the lactose content (r = 0.41, *P* < 0.001). Moreover, the lactating women with a college education or above (F_0.05, (2, 217)_ = 3.14, *P* = 0.04) and other (F_0.05, (1, 218)_ = 7.28, *P* = 0.01) as an occupation were more likely to have a higher protein concentration in breast milk. The lactating women from medium-income families had a higher total dry matter concentration (F_0.05, (2, 217)_ = 4.45, *P* = 0.01) and larger amount of energy (F_0.05, (2, 217)_ = 4.17, *P =* 0.02) in breast milk.

**Table 2:** The association of characteristics with concentration of breast milk macronurtrients among lactating women in south-central China

Characteristic	Protein(g/100 ml)					Fat(g/100 ml)					Lactose(g/100 ml)					Total dry matter(g/100 ml)					Energy(g/100Kal)				
	mean (SD)	*F*	*df*		*P*	mean (SD)	*F*	*df*		*P*	Mean (SD)	*F*	*df*		*P*	Mean (SD)	*F*	*df*		*P*	Mean (SD)	*F*	*df*		*P*
			*v*_*1*_	*v*_*2*_				*v*_*1*_	*v*_*2*_				*v*_*1*_	*v*_*2*_				*v*_*1*_	*v*_*2*_				*v*_*1*_	*v*_*2*_	
Lactation period																									
7 and below days	2.15(0.86)	25.8	5	214	< 0.001	2.60(0.95)	3.06	5	214	0.01	6.09(0.49)	10.81	5	214	< 0.001	11.93(1.10)	7.42	5	214	< 0.001	58.87(8.92)	3.49	5	214	0.01
8 to 15 days	1.66(0.85)					3.90(1.21)					6.48(0.32)					13.04(0.92)					68.22(13.41)				
16 to 30 days	1.71(0.83)					3.35(1.29)					6.55(0.25)					12.57(1.11)					67.15(9.23)				
31 to 90 days	1.17(0.22)					3.22(1.22)					6.56(0.31)					11.84(1.35)					62.00(11.46)				
91 to 180 days	0.95(0.15)					3.13(1.50)					6.64(0.25)					11.55(1.54)					60.25(13.80)				
181 ~ days	0.91(0.17)					3.05(1.79)					6.64(0.45)					11.4(1.90)					59.43(16.65)				
Age																									
25 and below years	1.31(0.69)	0.46	2	217	0.63	3.06(1.48)	0.68	2	217	0.51	6.50(0.43)	0.39	2	217	0.68	11.78(1.64)	1.48	2	217	0.23	60.27(14.73)	1.9	2	217	0.15
26 to 30 years	1.4(0.69)					3.3(1.42)					6.50(0.38)					12.13(1.43)					63.86(12.43)				
31 ~ years	1.43(0.93)					3.27(1.31)					6.56(0.37)					12.17(1.40)					63.78(11.60)				
Parity																									
Primipara	1.39(0.74)	0.7	1	218	0.4	3.24(1.44)	0.32	1	218	0.57	6.49(0.41)	2.75	1	218	0.1	12.05(1.53)	0.55	1	218	0.46	62.63(13.54)	0.1	1	218	0.75
Multipara	1.29(0.70)					3.1(1.38)					6.60(0.33)					11.86(1.46)					61.93(12.59)				
Delivery mode																									
Natural	1.31(0.54)	0.18	2	217	0.83	3.18(1.44)	1.52	2	217	0.22	6.48(0.46)	0.78	2	217	0.46	11.88(1.60)	1.91	2	217	0.15	60.99(14.50)	2.18	2	217	0.12
Forceps	1.42(0.24)					4.22(0.88)					6.40(0.44)					13.10(0.87)					72.00(7.92)				
Cesarean	1.36(0.77)					3.21(1.43)					6.53(0.35)					12.03(1.45)					62.94(12.42)				
Education																									
Junior or below	1.2(0.45)	3.14	2	217	0.04	3.14(1.57)	0.15	2	217	0.86	6.58(0.33)	0.86	2	217	0.42	11.79(1.62)	0.68	2	217	0.51	61.54(14.17)	0.26	2	217	0.77
Senior	1.33(0.66)					3.26(1.42)					6.49(0.43)					12.01(1.54)					63.17(13.03)				
College or above	1.51(0.90)					3.17(1.37)					6.49(0.40)					12.11(1.43)					62.13(13.25)				
Occupation																									
Housework	1.27(0.58)	7.28	1	218	0.01	3.21(1.42)	0.01	1	218	0.92	6.53(0.39)	0.79	1	218	0.37	11.91(1.53)	1.29	1	218	0.26	61.58(13.62)	1.55	1	218	0.21
Others	1.54(0.92)					3.19(1.46)					6.48(0.42)					12.15(1.49)					63.90(12.73)				
Family economic level																									
Low	1.26(0.52)	1.57	2	217	0.21	2.96(1.30)	1.98	2	217	0.14	6.52(0.44)	1.02	2	217	0.362	11.62(1.45)	4.45	2	217	0.01	59.64(11.87)	4.17	2	217	0.02
Middle	1.44(0.88)					3.38(1.48)					6.53(0.35)					12.27(1.49)					65.11(12.95)				
High	1.44(0.67)					3.25(1.51)					6.43(0.44)					12.08(1.57)					61.01(16.08)				

The significant test values of ANOVA will expressed by F_α,(ν1,ν2)_. F_α,(ν1,ν2)_: α = 0.05, ν_1_: degrees of freedom for between groups, ν_2_:Degrees of freedom for within group

**Fig. 2:**
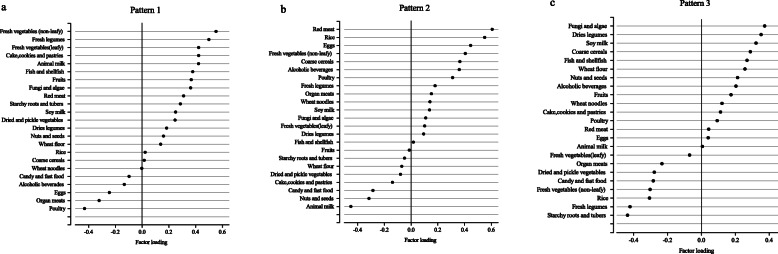
Factor loadings for three dietary patterns among lactating women in south-central China (*N* = 220).(Factor loadings of > |0.20| represent the foods which most strongly related to the identified factor). **a**: Factor loadings for pattern 1; **b**: Factor loadings for pattern 2; **c**: Factor loadings for pattern 3

Figure 2 shows the results of the principal component analysis to identify dietary patterns of lactating women. Three major dietary patterns were classified by factor analysis and explained 24.84% of the dietary intake variance. Dietary pattern 1 had high intake of fresh vegetables (leafy and non-leafy) and fresh legumes, but low intake of poultry, organ meats, and eggs. Dietary pattern 2 had high intake of red meat, cereals, and eggs, but lower intake of animal milk, nuts and seeds, candy, and fast food. Dietary pattern 3 had high intakes of fungi and algae, dried legumes, and soy milk, and lower intakes of starchy roots and tubers, fresh legumes, and rice.

Table 3 shows the breast milk composition by quartile categories of dietary patterns. The lactating women in the top quartile (Q4) of pattern 1 had lower concentrations of protein (F_0.05, (3, 216)_ = 12.04, *P* < 0.001) and total dry matter (F_0.05, (3, 216)_ = 2.88, *P* = 0.04) in breast milk, while those in pattern 2 had higher concentrations of protein (F_0.05, (3, 216)_ = 6.09, *P* < 0.001) and total dry matter (F_0.05, (3, 216)_ = 3.98, *P* = 0.01). There were significant differences in the lactose (F_0.05, (3, 216)_ = 7.32, *P* < 0.001) level of breast milk among the quartiles of pattern 1; a higher dietary pattern score was associated with a higher lactose concentration. There were no significant differences in the fat and energy levels of breast milk across quartiles of the three patterns.

**Table 3:** The breast milk macronutrients composition concentrations (Mean ± SD) of lactating women by quartile categories of dietary patterns

	Q1	Q2	Q3	Q4	F	*df*		*P*
						*v*_*1*_	*v*_*2*_	
*N*	55	55	55	55				
Pattern 1								
Protein	1.79(0.79)	1.43(0.83)	1.20(0.61)	1.06(0.42)	12.04	3	216	< 0.001
Fat	3.30(1.17)	3.23(1.51)	3.10(1.45)	3.18(1.58)	0.19	3	216	0.90
Lactose	6.32(0.43)	6.49(0.48)	6.61(0.31)	6.62(0.27)	7.32	3	216	< 0.001
Total dry matter	12.45(1.10)	12.07(1.66)	11.76(1.51)	11.71(1.64)	2.88	3	216	0.04
Energy	63.79(12.34)	63.00(13.46)	61.47(13.10)	61.44(14.51)	0.42	3	216	0.74
Pattern 2								
Protein	1.16(0.39)	1.29(0.72)	1.31(0.53)	1.71(1.03)	6.09	3	216	< 0.001
Fat	2.93(1.30)	3.15(1.41)	3.52(1.60)	3.21(1.36)	1.58	3	216	0.19
Lactose	6.50(0.45)	6.52(0.47)	6.56(0.28)	6.46(0.37)	0.56	3	216	0.64
Total dry matter	11.49(1.36)	11.86(1.62)	12.30(1.64)	12.33(1.30)	3.98	3	216	0.01
Energy	58.96(12.07)	61.69(13.08)	64.34(16.30)	64.71(10.74)	2.25	3	216	0.08
Pattern 3								
Protein	1.21(0.69)	1.39(0.72)	1.31(0.39)	1.57(0.97)	2.54	3	216	0.06
Fat	3.47(1.40)	3.06(1.50)	3.38(1.32)	2.90(1.45)	2.02	3	216	0.11
Lactose	6.52(0.33)	6.50(0.42)	6.46(0.49)	6.57(0.34)	0.76	3	216	0.52
Total dry matter	12.13(1.49)	11.85(1.55)	12.08(1.49)	11.93(1.55)	0.40	3	216	0.75
Energy	64.40(12.58)	61.31(13.14)	62.34(14.62)	61.65(13.00)	0.59	3	216	0.62

The significant test values of ANOVA will expressed by F_α,(ν1,ν2)_. F_α,(ν1,ν2)_: α = 0.05, ν_1_: degrees of freedom for between groups, ν_2_:Degrees of freedom for within groups

Table 4 presents the associations between dietary patterns and breast milk composition based on multivariate analysis. Overall, pattern 2 was positively associated with the concentrations of protein (B = 0.07, 95% CI 0.00, 0.15), total dry matter (B = 0.20, 95% CI 0.02, 0.38), and energy (B = 1.66, 95% CI 0.03, 3.30) in breast milk. Dietary patterns 1 and 3 were not significantly associated with the macronutrient concentrations in breast milk. Moreover, lactation period was negatively associated with the protein and total dry matter concentrations and positively associated with lactose in the multivariate linear regression model of associations between dietary patterns and breast milk concentration.

**Table 4:** Multivariable analysis of associations between dietary patterns and breast milk composition concentrations among lactating women in south-central China

	Protein		Fat		Lactose		Total dry matter		Energy	
	B (95% CI)	***P***	B (95% CI)	***P***	B (95% CI)	***P***	B (95% CI)	***P***	B (95% CI)	***P***
Pattern 1 (*N* = 220)										
Pattern 1	−0.07 (−0.15,0.02)	0.11	−0.04 (− 0.24,0.15)	0.67	0.05 (0.00,0.10)	0.07	− 0.11 (− 0.32,0.09)	0.27	− 0.34 (−2.20,1.52)	0.72
Lactation period	− 0.22 (0.27,-0.16)	< 0.001	− 0.01 (− 0.13,0.12)	0.90	0.07 (0.04,0.10)	< 0.001	− 0.18 (− 0.32,-0.05)	0.01	− 0.70 (−1.90,0.51)	0.26
Parity	–	–	–	–	0.08 (− 0.04,0.21)	0.17	–	–	–	–
Educational level	0.05 (−0.07,0.17)	0.40	–	–	–	–	–	–	–	–
Occupation	0.10 (−0.08,0.28)	0.29	–	–	–	–	–	–	–	–
Family economic status	–	–	–	–	–	–	0.24 (−0.04,0.53)	0.09	1.34 (−1.25,3.93)	0.31
Pattern 2 (*N* = 220)										
Pattern 2	0.07 (0.00, 0.15)	0.04	0.12 (−0.06,0.29)	0.18	0.03 (−0.02,0.08)	0.20	0.20 (0.02,0.38)	0.03	1.66 (0.03,3.30)	0.04
Lactation period	−0.23 (− 0.27, -0.18)	< 0.001	0.00 (− 0.12,0.11)	0.96	0.09 (0.06,0.12)	< 0.001	−0.20 (− 0.31,-0.08)	0.001	− 0.59 (−1.63,0.45)	0.26
Parity	–	–	–	–	0.09 (−0.03,0.21)	0.14	–	–	–	–
Educational level	0.04 (−0.08, 0.16)	0.50	–	–	–	–	–	–	–	–
Occupation	0.09 (−0.09, 0.27)	0.32	–	–	–	–	–	–	–	–
Family economic status	–	–	–	–	–	–	0.16 (−0.12,0.45)	0.25	0.82 (−1.76,3.39)	0.53
Pattern3 (*N* = 220)										
Pattern 3	0.05 (−0.02,0.13)	0.131	−0.15 (− 0.32,0.02)	0.09	0.03 (− 0.01,0.08)	0.18	−0.09 (− 0.26,0.09)	0.33	−0.91 (−2.50,0.68)	0.26
Lactation period	−0.23 (− 0.28,-0.19)	< 0.001	− 0.03 (− 0.14,0.07)	0.53	0.09 (0.06,0.11)	< 0.001	− 0.23 (− 0.34,-0.12)	< 0.001	−0.89 (−1.93,0.14)	0.09
Parity	–	–	–	–	0.09 (−0.03,0.21)	0.15	–	–	–	–
Educational level	0.05 (−0.06,0.17)	0.38	–	–	–	–	–	–	–	–
Occupation	0.10 (−0.08,0.28)	0.29	–	–	–	–	–	–	–	–
Family economic status	–	–	–	–	–	–	0.21 (−0.07,0.50)	0.13	1.23 (−1.33,3.78)	0.34

-: The variable was not included in the multivariable analysis. Significant predictors (*P* < 0.1) of breast milk macronurtrients composition in bivariate analyses were included as cofounding factors in the multivariable linear regression model. Lactation period, educational level and occupation were included in the multivariable linear regression model of associations between dietary patterns and protein concentration. Lactation period were included in the multivariable linear regression model of associations between dietary patterns and fat concentration. Lactation period and parity were included in the multivariable linear regression model of associations between dietary patterns and lactose concentration. Lactation period and family economic status were included in the multivariable linear regression model of associations between dietary patterns and total dry matter and energy concentration

## Discussion

We report here an association between maternal dietary pattern and breast milk macronutrient composition. The results showed that there are three major dietary patterns among lactating women in south-central China: dietary pattern 1 was mainly based on fresh vegetables and fresh legumes; dietary pattern 2 on red meat, cereals, and eggs; and dietary pattern 3 was based on fungi and algae, dried legumes, and soy milk. Moreover, the protein, total dry matter, and energy concentrations in breast milk were related to the dietary pattern.

Macronutrients such as protein, fat, and carbohydrates are the main nutrients in breast milk. In this study, the concentrations in breast milk within 12 months of delivery were 1.37 ± 0.73 g/100 mL for protein, 3.20 ± 1.43 g/100 mL for fat, and 6.51 ± 0.40 g/100 mL for lactose. Yang et al. reported that the concentrations in breast milk within 2 to 4 months of delivery in eastern China were 0.8 ± 0.1 g/100 mL for protein, 3.1 ± 1.4 g/100 mL for fat, and 7.2 ± 0.3 g/100 mL for lactose [8]. Cao et al. reported that the concentrations in breast milk within 15 months of delivery in Shanxi Province [18] were 1.5 ± 0.5 g/100 mL for protein, 5.9 ± 2.1 g/100 mL for fat, and 6.6 ± 1.5 g/100 mL for lactose. Huang et al. reported that the concentrations in breast milk within 9 months of delivery in Sichuan Province [19] were 1.14 (range 1.09–1.19) g/100 mL for protein, 4.1 ± 1.6 g/100 mL for fat, and 7.19 (range 6.78–7.42) g/100 mL for lactose. This suggests that the macronutrient concentrations in breast milk vary geographically (Table 5).

**Table 5:** Comparing to the breast milk macronutrients concentration in different areas of China

Area	***N***	Lactation period	Method	Concentration (Mean ± SD or Median (IQR),g/100 mL)
Protein				
Our study	220	0 to 12 months	Miris human milk analyzer	1.37 ± 0.73
East of China [9]^a^	90	2 to 4 months	Miris human milk analyzer	0.8 ± 0.1
Shanxi [20]	4317	0 to 15 months	Miris human milk analyzer	1.5 ± 0.5
Sichuan [21]	60	0 to 9 months	Miris human milk analyzer	1.14 (1.09,1.19)
Fat				
Our study	220	0 to 12 months	Miris human milk analyzer	3.2 ± 1.43
East of China	90	2 to 4 months	Miris human milk analyzer	3.1 ± 1.4
Shanxi	4317	0 to 15 months	Miris human milk analyzer	5.9 ± 2.1
Sichuan	60	0 to 9 months	Miris human milk analyzer	4.1 ± 1.6
Lactose				
Our study	220	0 to 12 months	Miris human milk analyzer	6.51 ± 0.40
East of China	90	2 to 4 months	Miris human milk analyzer	7.2 ± 0.3
Shanxi	4317	0 to 15 months	Miris human milk analyzer	6.6 ± 1.5
Sichuan	60	0 to 9 months	Miris human milk analyzer	7.19 (6.78,7.42)

^a^: The breast milk composition represented three cities from east of China, including Beijing, Guangzhou and Suzhou

In addition, the concentrations of protein, fat, and lactose differed significantly with the stage of lactation in bivariate analyses in our study. The lactation stage is the factor that affects milk composition most strongly; this has been shown in several studies [8, 20]. In our multivariate analysis, lactation period was negatively associated with the protein concentration, but positively associated with lactose, which is consistent with previous studies [5, 8]. However, no association was found between the fat concentration and lactation period in the multivariate analysis, which is inconsistent with other reports that the fat content declined with the lactation period [8].

Many recent studies have reported associations between the maternal diet and milk macronutrient composition. Some studies reported a positive effect of maternal diet on breast milk composition, while others showed the opposite [21]. In our study, the protein concentration and energy in breast milk were positively related to dietary pattern 2. Pattern 2 was characterized by high intakes of red meat, cereals, and eggs. Red meat is a high-energy-density food that contains large amounts of saturated fatty acids and cholesterol, so a high intake may lead to excessive energy intake [22]. Venus et al. reported a correlation between the total protein concentration in human milk and maternal daily energy and fat intakes [23]. Protein is important for physical growth and brain development of neonates, especially premature infants who need more protein for growth and development; however, the breast milk protein content in these mothers decreased more with the lactation period than in term infants [24]. Therefore, a rational dietary pattern for lactating women will be an effective way to improve infant nutrition.

The total dry matter content of breast milk is important for promoting infant growth and development. Wu et al. reported that the higher the total dry matter content of breast milk, the greater the height of the infant [25]. However, few studies have focused on the association of total dry matter concentration in breast milk with dietary intake. We found that dietary pattern 2 (high intake of red meat, cereals, and eggs) was associated with the total dry matter content of breast milk. Therefore, the maternal dietary pattern may influence the total dry matter content of breast milk.

Fat is a major source of energy in human milk. Several studies found positive associations between dietary fatty acid intake and the fatty acid concentration in breast milk [10, 21, 26], while others reported no such association [27, 28]. In the current study, the pattern was not significantly associated with the fat concentration in breast milk. Lactose is the principal carbohydrate in breast milk. According to Wang et al., the lactose concentration in the breast milk of vegetarian lactating women was significantly lower than that of meat-eaters [29]. However, the lactose concentration in breast milk was not associated with the maternal diet in our study.

Previous studies have shown that economic status is strongly associated with maternal diet, where family income influences the variety and quantity of food intake. Li et al. reported that family economic status was related to fruit intake in mothers [30], while Wang et al. reported that family economic status was positively correlated with the dietary diversity of mothers [31]. However, in this study multivariate analysis showed no association between dietary patterns and family economic status. This finding may be attributed to the similar demographic characteristics of participants, as they were all recruited from one city. Therefore, the effect of economic status on maternal dietary patterns should be considered in future studies.

This study had several highlights and limitations. First, to our knowledge, this is the first study of the macronutrient composition of breast milk of lactating women and its association with dietary pattern in south-central China, so the findings provide a valuable basis for improving maternal nutrition and infant feeding in this region. Second, we performed a principal component analysis to analyse dietary patterns, taking into account overall food intake and food interactions. Nevertheless, this study also had shortcomings. First, we used the 24 h recall method to collect dietary information, which may be subject to recall bias. Second, a small number of lactating women was found in the local communities and some were reluctant to enrol in the study. In total, only 220 lactating women were recruited. The sample size is too small to represent the general population, which may limit the generalizability of our findings. Third, we analysed only the association between macronutrients in breast milk and the dietary pattern. While micronutrients such as vitamins and minerals are also important components of breast milk, their contents may be affected by dietary intake [32–35], so their associations need to be clarified in future studies.

## Conclusion

Three major dietary patterns were identified among lactating women in south-central China. Lactation period was an important factor affecting milk composition and a dietary pattern with high intake of red meat, cereals, and eggs was associated with higher protein, total dry matter, and energy contents in breast milk. These findings show that the dietary patterns of lactating women can affect breast milk macronutrient composition and provide a foundation for improving child health.

## Supplementary information

**Additional file 1: Table S1**. Food grouping used in the dietary pattern analyses.

**Additional file 2.** Lactating women dietary questionnaire.

## Data Availability

The datasets used and/or analyzed during the current study are available from the corresponding author on reasonable request.

## Abbreviations

N: Number of participants
ANOVA: Variance analyses
SD: Standard deviation
IQR: interquartile range
B: Unstandardized coefficients
CI: Confidence interval.
