# Supplementary material for: Dietary patterns and their association with breast milk macronutrient composition among lactating women
Source: Int Breastfeed J. 2020 Jun 5;15:52. doi: 10.1186/s13006-020-00293-w (PMC7273657; doi:10.1186/s13006-020-00293-w)
Supplement: Supplementary file 1 — Additional file 1: Table S1. Food grouping used in the dietary pattern analyses. [file 13006_2020_293_MOESM1_ESM.doc]

Table S1

| Food groups | Food items |
| --- | --- |
| Rice | Rice, rice flour, rice porridge |
| Wheat noodles | Wheat noodles |
| Wheat flour | Wheat flour, steamed buns, fried sticks, dumplings, wheat meal, breads |
| Coarse cereals | Maize, millet |
| Cakes, cookies and pastries | Cookies, moon cake, cake |
| Starchy roots and tubers | Potato, yam, taro, lotus root, cassava |
| Dried legumes | Soybean flour, dried beans, tofu, tofu products |
| Fresh legumes | French bean, bean sprouts, sword bean |
| Nuts and seeds | Sesame, watermelon, pumpkin, sunflower, lotus seeds, peanuts, walnuts, pine-nuts |
| Fresh vegetables, non-leafy | Loofah, cucumber, pumpkin, pepper, white gourd, bitter gourd, radish, carrot, tomato, eggplant |
| Fresh vegetables, leafy | Water spinach, cabbage, day lily, leeks, lettuce |
| Dried and pickled vegetables | Dried vegetables, salted vegetables, Chinese sauerkraut |
| Fungi and algae | Kelp, seaweed, mushroom, shiitakes, enoki |
| Fruits | Apple, banana, grape, watermelon, pear, orange, date, cantaloupe, longan, pomegranate and et al. |
| Red meat | Beef, lamb, pork and pork products |
| Organ meats | Liver, kidney, large intestine, blood |
| Poultry | Chicken, duck, goose |
| Egg | Duck eggs, chicken eggs |
| Fish and shellfish | Fish, shrimp, shellfish, crab |
| Soy milk | Soy milk |
| Animal milk | Milk, goat's milk, dairy products |
| Candy and fast foods | Chocolate, honey, sugar, candies, mixed congee, instant noodles, potato chips |
| Alcoholic beverages | Coca-cola, sprite, fruit and vegetable drink,  fruits juice, tea, grape wine, sweet wine; |
