# Supplementary material for: Dietary patterns and their association with breast milk macronutrient composition among lactating women
Source: Int Breastfeed J. 2020 Jun 5;15:52. doi: 10.1186/s13006-020-00293-w (PMC7273657; doi:10.1186/s13006-020-00293-w)
Supplement: Supplementary file 2 — Additional file 2. Lactating women dietary questionnaire. [file 13006_2020_293_MOESM2_ESM.docx]

English Version

Lactating Women Dietary Questionnaire

1. Home Address: ____________ Province ____________City ____________County

________ Town ________ Village __________Household Number

1. Name (C1) : ______________________________________
2. ID Number (C2) : __________________________________
3. Birth Date (C3) : ____ Year ____ Month ____ Day
4. [Delivery](javascript:showjdsw('jd_t','j_')) Date (C4) : ____ Year ____ Month ____ Day
5. Telephone Number (C5) : ____________________
6. Education (C6) : ____

a. [Illiteracy](http://dict.cnki.net/dict_result.aspx?searchword=%e6%96%87%e7%9b%b2&tjType=sentence&style=&t=illiteracy) b. Primary c. [Junior](http://dict.cnki.net/dict_result.aspx?searchword=%e5%88%9d%e4%b8%ad&tjType=sentence&style=&t=junior+high+school) d. Senior d. [junior college](http://dict.cnki.net/dict_result.aspx?searchword=%e5%a4%a7%e4%b8%93&tjType=sentence&style=&t=junior+colleges) e. College or above

1. Occupation (C7) :____

a: Administration b. Technician c. Staff d. Business services e. Animal husbandry and fishery e. [Operator](http://dict.cnki.net/dict_result.aspx?searchword=%e6%93%8d%e4%bd%9c%e4%ba%ba%e5%91%98&tjType=sentence&style=&t=operator) f. [Soldiers](http://dict.cnki.net/dict_result.aspx?searchword=%e5%86%9b%e4%ba%ba&tjType=sentence&style=&t=soldiers) g. Housework i. Others

1. Family Income in Past Year. __________ Yuan. (C8)
2. [Delivery mode](http://dict.cnki.net/dict_result.aspx?searchword=%e5%88%86%e5%a8%a9%e6%96%b9%e5%bc%8f&tjType=sentence&style=&t=delivery+mode) (C9).________
3. Natural b. Forceps c. Cesarean
4. The Frequency of [Pregnancy](http://dict.cnki.net/dict_result.aspx?searchword=%e6%80%80%e5%ad%95&tjType=sentence&style=&t=pregnancy) (Including [abortion](http://dict.cnki.net/dict_result.aspx?searchword=%e6%b5%81%e4%ba%a7&tjType=sentence&style=&t=abortion)). ________ (C10)
5. The Frequency of [Delivery](http://dict.cnki.net/dict_result.aspx?searchword=%e6%80%80%e5%ad%95&tjType=sentence&style=&t=pregnancy)._________ (C11)

Interview Date (D0) : ____ Year ____ Month ____ Day

Interviewer Name (S0) :______________________

Table 1. The First Day Record of Food Consumption (gram)

| Mealtime  M1a | Meal Location  M1b | Food Name | Ingredient Name | Ingredient Code  M1c | Weight (g)  M1d |
| --- | --- | --- | --- | --- | --- |
|  |  |  |  |  |  |
|  |  |  |  |  |  |
|  |  |  |  |  |  |
|  |  |  |  |  |  |
|  |  |  |  |  |  |
|  |  |  |  |  |  |
|  |  |  |  |  |  |
|  |  |  |  |  |  |
|  |  |  |  |  |  |
|  |  |  |  |  |  |
|  |  |  |  |  |  |
|  |  |  |  |  |  |
|  |  |  |  |  |  |
|  |  |  |  |  |  |
|  |  |  |  |  |  |
|  |  |  |  |  |  |
|  |  |  |  |  |  |
|  |  |  |  |  |  |
| M1a: 1=Breakfast 2= Snack in morning 3=Lunch 4=Snack in afternoon 5=Dinner 6=Snack in night  M1b: 1=Home 2=Workplace 3=Restaurant 4= Friend’s House 5=Festival 6=Others | | | | | |

Interview Date (D1) : ____ Year ____ Month ____ Day

Interviewer Name (S1) :______________________

Table 2. The Second Day Record of Food Consumption (gram)

| Mealtime  M1a | Meal Location  M1b | Food Name | Ingredient Name | Ingredient Code  M1c | Weight (g)  M1d |
| --- | --- | --- | --- | --- | --- |
|  |  |  |  |  |  |
|  |  |  |  |  |  |
|  |  |  |  |  |  |
|  |  |  |  |  |  |
|  |  |  |  |  |  |
|  |  |  |  |  |  |
|  |  |  |  |  |  |
|  |  |  |  |  |  |
|  |  |  |  |  |  |
|  |  |  |  |  |  |
|  |  |  |  |  |  |
|  |  |  |  |  |  |
|  |  |  |  |  |  |
|  |  |  |  |  |  |
|  |  |  |  |  |  |
|  |  |  |  |  |  |
|  |  |  |  |  |  |
|  |  |  |  |  |  |
| M2a: 1=Breakfast 2= Snack in morning 3=Lunch 4=Snack in afternoon 5=Dinner 6=Snack in evening  M2b: 1=Home 2=Workplace 3=Restaurant 4= Friend’s House 5=Festival 6=Others | | | | | |

Interview Date (D2) : ____ Year ____ Month ____ Day

Interviewer Name (S2) :______________________

Table 3. The Third Day Record of Food Consumption (gram)

| Mealtime  M1a | Meal Location  M1b | Food Name | Ingredient Name | Ingredient Code  M1c | Weight (g)  M1d |
| --- | --- | --- | --- | --- | --- |
|  |  |  |  |  |  |
|  |  |  |  |  |  |
|  |  |  |  |  |  |
|  |  |  |  |  |  |
|  |  |  |  |  |  |
|  |  |  |  |  |  |
|  |  |  |  |  |  |
|  |  |  |  |  |  |
|  |  |  |  |  |  |
|  |  |  |  |  |  |
|  |  |  |  |  |  |
|  |  |  |  |  |  |
|  |  |  |  |  |  |
|  |  |  |  |  |  |
|  |  |  |  |  |  |
|  |  |  |  |  |  |
|  |  |  |  |  |  |
|  |  |  |  |  |  |
| M3a: 1=Breakfast 2= Snack in morning 3=Lunch 4=Snack in afternoon 5=Dinner 6=Snack in night  M3b: 1=Home 2=Workplace 3=Restaurant 4= Friend’s House 5=Festival 6=Others | | | | | |

Interview Date (D3) : ____ Year ____ Month ____ Day

Interviewer Name (S3) :______________________

Chinese Version

乳母膳食营养状况调查表

1. 家庭住址:_______________________省_____________________市/县

_________________ 区/乡/镇_________________路/街/村

_________________小区_____________栋_____________室

2.姓名（C1）：____________________________

3.身份证号（ C2）： _____________________________

4.出生日期（ C3）：________ 年________ 月________ 日（阳历）

5.分娩日期（ C4）：________ 年________ 月________ 日（阳历）

6.联系方式（ C5）：__________________________________

7. 文化程度（ C6） : ____
①文盲 ②小学 ③初中 ④高中/中专 ⑤大专/职大 ⑥大学及以上

8. 职业（ C7）: ____
①机关/企事业单位负责人 ②专业技术人员 ③办事人员和有关人员④商业服务业人员 ⑤农林牧渔水利业生产人员 ⑥生产运输设备操作人员 ⑦军人 ⑧家务 ⑨其他

9.去年，全家的总收入是____元？（ C8）

10.采用了哪种分娩（生孩子）方式 ？____（ C9）
①自然分娩 ②人工辅助分娩（如侧切、使用产钳等） ③剖腹产

11.怀孕了多少次 (包括流产) ？____（ C10）

12.生产了几次？ ____（ C11）

调查日期(D0)：____ 年____月____ 日

调查员(S0)： _____________________

乳母第1天 24 小时膳食回顾询问表 3-1

| 进餐时间  M1a | 进餐地点  M1b | 食物名称 | 原料名称 | 原料编码  M1c | 原料重量(g)  M1d |
| --- | --- | --- | --- | --- | --- |
|  |  |  |  |  |  |
|  |  |  |  |  |  |
|  |  |  |  |  |  |
|  |  |  |  |  |  |
|  |  |  |  |  |  |
|  |  |  |  |  |  |
|  |  |  |  |  |  |
|  |  |  |  |  |  |
|  |  |  |  |  |  |
|  |  |  |  |  |  |
|  |  |  |  |  |  |
|  |  |  |  |  |  |
|  |  |  |  |  |  |
|  |  |  |  |  |  |
|  |  |  |  |  |  |
|  |  |  |  |  |  |
|  |  |  |  |  |  |
|  |  |  |  |  |  |
| 注： M1a: 1=早餐 2=上午小吃 3=午餐 4=下午小吃 5=晚餐 6=晚上小吃 M1b: 1=在家 2=单位/学校 3=饭馆/摊点 4=亲戚/朋友家 5=节日/庆典 6=其他 | | | | | |

调查日期(D1)：____ 年____月____ 日

调查员(S1)： ______________________

乳母第2天 24 小时膳食回顾询问表 3-2

| 进餐时间  M2a | 进餐地点  M2b | 食物名称 | 原料名称 | 原料编码  M2c | 原料重量(g)  M2d |
| --- | --- | --- | --- | --- | --- |
|  |  |  |  |  |  |
|  |  |  |  |  |  |
|  |  |  |  |  |  |
|  |  |  |  |  |  |
|  |  |  |  |  |  |
|  |  |  |  |  |  |
|  |  |  |  |  |  |
|  |  |  |  |  |  |
|  |  |  |  |  |  |
|  |  |  |  |  |  |
|  |  |  |  |  |  |
|  |  |  |  |  |  |
|  |  |  |  |  |  |
|  |  |  |  |  |  |
|  |  |  |  |  |  |
|  |  |  |  |  |  |
|  |  |  |  |  |  |
|  |  |  |  |  |  |
| 注： M2a: 1=早餐 2=上午小吃 3=午餐 4=下午小吃 5=晚餐 6=晚上小吃 M2b: 1=在家 2=单位/学校 3=饭馆/摊点 4=亲戚/朋友家 5=节日/庆典 6=其他 | | | | | |

调查日期(D2)：____ 年____月____ 日

调查员(S2)： ______________________

乳母第3天 24 小时膳食回顾询问表 3-3

| 进餐时间  M3a | 进餐地点  M3b | 食物名称 | 原料名称 | 原料编码  M3c | 原料重量(g)  M3d |
| --- | --- | --- | --- | --- | --- |
|  |  |  |  |  |  |
|  |  |  |  |  |  |
|  |  |  |  |  |  |
|  |  |  |  |  |  |
|  |  |  |  |  |  |
|  |  |  |  |  |  |
|  |  |  |  |  |  |
|  |  |  |  |  |  |
|  |  |  |  |  |  |
|  |  |  |  |  |  |
|  |  |  |  |  |  |
|  |  |  |  |  |  |
|  |  |  |  |  |  |
|  |  |  |  |  |  |
|  |  |  |  |  |  |
|  |  |  |  |  |  |
|  |  |  |  |  |  |
|  |  |  |  |  |  |
| 注： M3a: 1=早餐 2=上午小吃 3=午餐 4=下午小吃 5=晚餐 6=晚上小吃 M3b: 1=在家 2=单位/学校 3=饭馆/摊点 4=亲戚/朋友家 5=节日/庆典 6=其他 | | | | | |

调查日期(D3)：____ 年____月____ 日

调查员(S3)： ______________________
